# Metasurface-Loaded Biodegradable Mobile Phone Back Cover for Enhanced Radiation Performance

**DOI:** 10.3390/ma18040730

**Published:** 2025-02-07

**Authors:** Juin Acharjee, Jawad Ali, Muhammad Uzair, Thipamas Phakaew, Prayoot Akkaraekthalin, Yaowaret Maiket, Rungsima Yeetsorn, Suramate Chalermwisutkul

**Affiliations:** 1 High Frequency Systems Laboratory, King Mongkut’s University of Technology North Bangkok, Bangkok 10800, Thailand; juin.ece@gmail.com (J.A.); jawad.ali@email.kmutnb.ac.th (J.A.); muhammad.uzair@email.kmutnb.ac.th (M.U.); thipamas.p@email.kmutnb.ac.th (T.P.); 2 Department of ECE, ST. Thomas’ College of Engineering and Technology, Kolkata 700023, India; 3 Faculty of Engineering, King Mongkut’s University of Technology North Bangkok, Bangkok 10800, Thailand; prayoot.a@eng.kmutnb.ac.th; 4 Chemical Process Engineering Technology, Faculty of Engineering and Technology, King Mongkut’s University of Technology North Bangkok, Rayong 21120, Thailand; yaowaret.m@eat.kmutnb.ac.th; 5 Materials and Production Engineering Program, The Sirindhorn International Thai–German Graduate School of Engineering, King Mongkut’s University of Technology North Bangkok, Bangkok 10800, Thailand; rungsima.y@tggs.kmutnb.ac.th

**Keywords:** biodegradable substrate, metasurface, specific absorption rate, MIMO antenna, bandwidth enhancement

## Abstract

This article introduces a novel biodegradable metasurface-loaded mobile phone back cover designed to reduce electromagnetic exposure and enhance antenna performance. The cover operates across the low GHz band (2–8 GHz) and the millimeter-wave band (22–25.6 GHz), utilizing polylactic acid as an eco-friendly substrate. Integrated with a six-port multiple-input multiple-output (MIMO) antenna system, the cover achieves port isolation above 20 dB in both bands. Specific absorption rate (SAR) analysis, performed using a human head model, shows significant reductions in electromagnetic exposure—61.1% in the low GHz band (from 1.06 W/kg to 0.412 W/kg) and 55% in the millimeter wave band (from 2.061 W/kg to 0.917 W/kg). Additionally, the metasurface cover enhances antenna gain and increases impedance bandwidth by 20% in the low GHz band and 8.3% in the millimeter-wave band. A comparative study highlights superior SAR reduction and bandwidth improvement of a metasurface on a biodegradable substrate over one on a silicone substrate. Prototypes of the MIMO antenna and the proposed cover were fabricated and tested, revealing strong alignment between simulated and measured results. These findings highlight the potential of biodegradable metasurface-based covers to deliver high-performance, sustainable solutions for mobile communication devices.

## 1. Introduction

The rapid growth of electronic waste poses a significant challenge to the environment, as the majority of these toxic materials are either deposited directly into landfills or incinerated due to the difficulties and high costs associated with recycling [1]. In response to this environmental concern, there has been growing interest in the development of biodegradable and biocompatible materials for electronic applications, which aim to mitigate the impact of e-waste by offering more sustainable alternatives to traditional materials [2]. Polylactic acid (PLA), a biodegradable polyester derived from renewable sources such as corn and sugarcane, has emerged as a promising candidate due to its favorable properties, including light weight, affordability, and excellent plasticity and rigidity [3]. The diverse range of feedstocks for PLA production ensures flexibility and reduces dependence on specific geographic regions. As a biopolymer, PLA decomposes into natural elements when composted, unlike conventional plastics that persist in the environment for centuries.

The production of PLA is environmentally friendly because the cultivation of its feedstock absorbs CO_2_ from the atmosphere. Since CO_2_ is released back at the end of PLA’s lifecycle, the overall process supports carbon neutrality and aligns with net-zero emission goals [4]. PLA offers several advantages, including mechanical strength, optical clarity, biocompatibility, and a low processing temperature, making it a versatile, eco-friendly material for applications such as food packaging, biomedical devices, and 3D printing [5,6]. With its well-established production infrastructure and energy-efficient manufacturing processes, PLA has become a dominant biopolymer in the market. In 2021, it held the largest market share for biodegradable bioplastics, significantly outperforming competitors like polyhydroxyalkanoates (PHAs) in production capacity [7]. Despite its advantages, PLA has limitations for certain applications due to its brittleness and poor ductility. To address these issues, PLA can be combined with other eco-friendly materials to form composites that enhance its ductility [5]. Polybutyrate adipate terephthalate (PBAT), with superior ductility, heat resistance, impact capacity, and biodegradability, presents a promising alternative. However, PBAT’s production infrastructure is less developed than that of PLA, and its mechanical strength is lower.

Beyond its environmental benefits, PLA’s advantageous electromagnetic properties have attracted attention for applications in modern wireless communication systems [8]. The widespread adoption of wireless technologies, particularly in mobile devices, has created a continuous demand for advancements in antenna performance and enhancements in user safety for personal communications. Next-generation communication systems demand mobile antennas with smaller dimensions, larger bandwidth, stable gain, and reduced specific absorption rate (SAR) [9]. Conventional microstrip patch antennas (MPAs) face challenges in meeting these requirements. Researchers have explored various techniques to overcome these limitations as reported in the literature [10,11,12,13,14,15,16,17,18,19,20]. In [10], a pin-loaded patch antenna is fed by a dual-mode substrate-integrated waveguide to enhance the bandwidth and to achieve stable antenna gain. The addition of a ground stub to an inverted L antenna [11] and embedding a rectangular patch loaded with shorting vias [12] are reported techniques for significant improvements in the bandwidth and gain of antennas. In [13], a combination of multiple parasitic patches and shorting vias is used with a conventional triangular patch antenna to achieve bandwidth enhancement. In [14], contactless varactor diodes and shorting vias are employed to bring the TM_10_ mode closer to the TM_30_ mode, effectively broadening the bandwidth. Numerous studies have been conducted to reduce the SAR of antennas [15,16,17,18,19,20]. Several techniques have been reported for reducing antenna SAR, including the use of fractal geometry, meandering slits, and defected ground structures [15]; integrating metamaterial structures in the superstrate layer [16]; canceling total current equalization (TCE) generated by the predicted total current mode [17]; and adjusting the antenna surface current [18]. In [19], EBG structures are used to significantly reduce SAR and radiation toward the human head. The use of novel multiport clustering is another technique that can reduce the SAR of an antenna by over 50% compared to conventional designs while maintaining high efficiency [20].

As the increasing demand for high-speed data in modern wireless communications led to significant congestion in traditional frequency bands, there is a growing interest in utilizing higher frequency ranges, such as the millimeter-wave (mmWave) band, alongside the sub-6 GHz bands [9]. Antennas operating in these bands must meet stringent requirements, including broad bandwidth, efficient radiation patterns, and reduced specific absorption rate (SAR), to ensure optimal performance and user safety. Achieving both bandwidth enhancement and SAR reduction in the mmWave band is particularly critical for supporting the data-intensive needs of next-generation communication systems. An attempt to fulfill the requirements in both low GHz and mmWave bands often results in complex antenna structures and poses challenges for integration, miniaturization, and cost-effectiveness. Nowadays, the use of multiple-input multiple-output (MIMO) antennas in mobile communications has become widespread due to their benefits, including increased data throughput, enhanced reliability, reduced interference, and improved coverage and range [21,22,23]. However, their use is often limited to specific frequency bands, such as sub-6 GHz, mid-band, or mmWave bands, with a limited number of antenna elements. Therefore, modern wireless standards demand antennas capable of operating across multiple bands simultaneously with the maximum use of antenna elements with optimal performance. Using multiple antennas in mobile phones and their prolonged usage also raises concerns about non-ionizing radiation exposure to the human body [24,25], leading to health concerns [26,27,28]. Therefore, the designer should consider various key parameters to develop antenna systems suitable for future mobile communications.

Metasurfaces (MTSs), two-dimensional arrays of subwavelength-scale artificial structures, are widely used to enhance antenna performance by increasing bandwidth and gain while reducing SAR [29,30,31]. Phase-gradient metasurfaces (PGMs) reduce radar cross-section (RCS) and enhance patch antenna gain [29], while combining reactive impedance surfaces (RISs) with polarization conversion metasurfaces (PCMs) enables antenna miniaturization, bandwidth enhancement, and broadband RCS reduction [30]. Properly placing MTSs near patch antennas improves gain and focuses electromagnetic waves [31,32]. These techniques have been applied to reduce SAR and enhance performance in 5G mobile phones [31], but they often fail to meet modern requirements such as multi-band operation, compact size, and consistent SAR reduction across bands [33]. To address these challenges, designing multi-band MTS within limited space is essential. Using MTS as an external layer increases antenna size, but integrating MTS into the phone case as a substrate layer offers a space-efficient alternative. This research builds on [31], focusing on multi-band antenna modules for high-speed connectivity while incorporating eco-friendly materials as MTS substrates to enhance performance and sustainability.

In this work, a novel approach is presented that integrates a metasurface layer on a biodegradable PLA-based mobile phone case. This innovative design aims to achieve the following:

- Enhance antenna bandwidth by improving impedance matching for both useful frequency bands;
- Reduce SAR by minimizing back radiation from the antenna, thereby decreasing the electromagnetic energy absorbed by the user for both bands;
- Promote sustainability by using PLA as the primary material for the mobile phone case.

This biodegradable mobile back cover offers multiple benefits, including low thermal expansion, strong and durable protection with excellent mechanical properties, and environmental sustainability, through its biodegradability and reduced carbon footprint [34]. To explore the potential of biodegradable materials in mobile communication applications, polylactic acid (PLA) has been characterized for its dielectric properties. This assessment evaluates its suitability as a substrate for a mobile phone case integrated with a metasurface, designed to reduce the specific absorption rate (SAR) and enhance antenna performance. Following the characterization, a prototype of a PLA-based metasurface mobile back cover was fabricated. The antenna performance with the PLA-based metasurface cover was compared to that of a metasurface cover made from traditional plastics such as silicone. To the best of the authors’ knowledge, this is the first attempt to use an MTS-loaded biodegradable material as a mobile cover to reduce SAR and enhance antenna system performance.

## 2. Materials and Methods

### 2.1. Polylactic Acid and Its Properties

Polylactic acid (PLA) is a thermoplastic aliphatic polyester derived from renewable resources [35,36]. Its molecular structure consists of repeating units of lactic acid, which can be polymerized through various processes to form PLA. The polymer chain’s configuration, either in a semi-crystalline or amorphous state, significantly influences the material’s mechanical properties and thermal behavior [37,38,39]. Understanding the molecular structure of PLA is crucial for tailoring its characteristics to meet specific application requirements in wireless communication technologies. PLA is renowned for its biodegradability and biocompatibility, making it an environmentally friendly alternative to traditional plastics. Its mechanical properties, such as tensile strength and modulus, are comparable to those of conventional petroleum-based plastics, which makes it suitable for a wide range of applications. Additionally, PLA exhibits good thermal stability and can be processed using standard manufacturing techniques, including injection molding and 3D printing [40,41,42]. These properties make PLA an attractive option for developing sustainable components in wireless communication devices, reducing the ecological footprint of the industry.

PLA is known for its susceptibility to thermal degradation and loss of mechanical strength under high-temperature conditions. However, the typical usage conditions of mobile phones, primarily in indoor environments or shaded areas, mitigate these concerns to an extent. For a mobile phone cover made of PLA, durability is largely influenced by environmental factors such as temperature, UV exposure, and humidity. As mobile phones are seldom subjected to prolonged exposure to direct sunlight or extreme temperatures during regular use, it is reasonable to expect that a PLA-based mobile phone case could maintain its structural integrity for approximately 1–3 years under normal conditions.

### 2.2. Fabrication of PLA Samples

**Figure 1:**
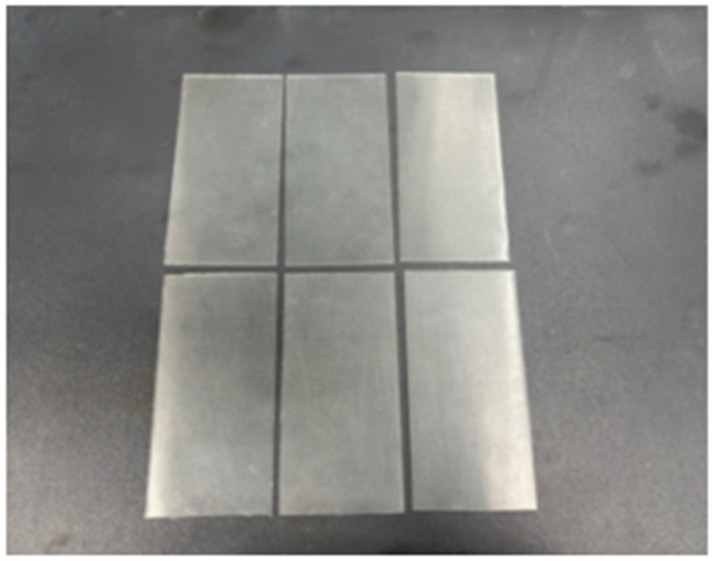
Fabricated PLA samples.

The production of PLA involves several steps, starting with the fermentation of raw materials like corn or sugarcane to produce lactic acid. This lactic acid is then polymerized through condensation or ring-opening polymerization to form PLA. Advances in production technologies have improved the efficiency and cost-effectiveness of PLA manufacturing, enabling its broader adoption in various sectors. Moreover, ongoing research aims to enhance the properties of PLA through copolymerization and blending with other materials, further expanding its potential applications in wireless communications [43,44]. Initially, the samples were fabricated using a compression molding machine of the company SIEMENS^TM^, Erlangen, Germany. The samples in this work were made from polylactic acid, PLA (Ingeo™ Biopolymer 4043D from NatureWorks, Plymouth, MN, USA). The sample batch was preheated at 175 °C under a pressure of 1 MPa for 3 min, then subjected to 10 MPa pressure at the same temperature for an additional 5 min. Following the heating phase, a pressure cooling process was carried out for 10 min. Ultimately, each specimen, sized 80 × 170 × 3 mm, was developed as shown in Figure 1.

### 2.3. Characterization of PLA Substrate

The PLA sheets fabricated using the process described in Section 2.2 are used as the substrate of the MTS in this work. To use this material, it is important to determine the electrical properties of the material for accurate simulations. The fabricated PLA substrate was characterized for both low GHz and mmWave bands. In addition, a conventional petroleum-based plastic mobile back cover was also characterized to make a comparison between both materials. Since no previous reports exist on the electrical properties of these materials for mmWave frequencies, characterization of both the materials was conducted to determine their electrical properties. The T-resonator method [45] was used to extract the material’s relative dielectric constant (*ε_r_*) and loss tangent (*tan* *δ*) for the frequency ranges of 2–8 GHz and 24–28 GHz. The T-resonator comprises a microstrip open stub and feeding lines [45]. The length (*L_r_*) of the open stub is determined using the following quarter wave resonator (λ_g_/4) design equation:

(1) $$L_{r}=\frac{n.c}{4f\sqrt{\varepsilon_{eff}}}$$

where *n* is the order of the resonance (*n* = 1, 2, 3, ….), *c* is the velocity of light, *f* is the resonant frequency, and *ε_eff_* is the effective dielectric constant of the microstrip line, which depends on the dielectric constant of the substrate material as well as the microstrip line’s geometry and configuration. By designing the T-resonator and measuring its resonance frequencies for various modes, the effective dielectric constant and loss tangent of the material can be calculated using Equations (2)–(4) as follows:

(2) $$\varepsilon_{eff\;}(n)={\left[\frac{nc}{4(L_{r}+\frac{w_{r}}{2}+l_{eo}-d_{2})\;f(n)}\right]}^{2}$$

where *L_r_* and *w_r_* are the length and width of the stub, respectively. *l_eo_* and *d*_2_ are the correction factors for the effects of the open end and the T-junction [30]. After calculating *ε_eff_*, *ε_r_* can be calculated using (3) [46].

(3) $$\varepsilon_{eff}=\frac{\varepsilon_{r}+1}{2}+\frac{\varepsilon_{r}-1}{2\sqrt{1+12(\frac{h_{r}}{w_{r}})}}$$

The loss tangent (*tan δ*) of the material is extracted from the unloaded quality factors (*Q_d_*) and dielectric constant of the material using Equation (4) [46].

(4) $$tan\;\delta =\frac{\varepsilon_{eff}(\varepsilon_{r}-1)}{Q_{d}\varepsilon_{r\;}(\varepsilon_{eff}-1)}$$

The calculated dielectric constant and loss tangent for the fabricated PLA substrate are *ε_r_* = 2.54 and *tan δ* = 0.0071, respectively. In contrast, for the conventional plastic case, these values are *ε_r_* = 2.81 and *tan δ* = 0.0074. All calculated values represent the average over the band of interest.

## 3. Results and Discussion

### 3.1. Design and Characterization of the MTS Unit Cell

**Figure 2:**
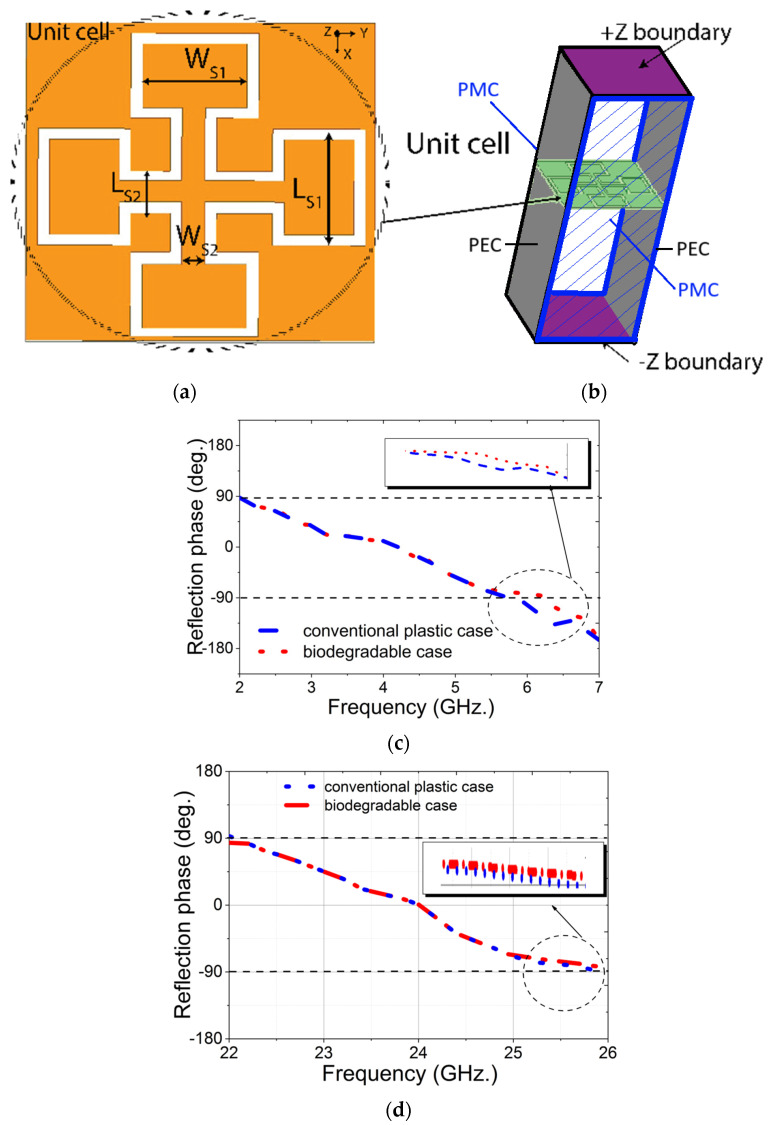
(**a**) Structure of the MTS unit cell (dimensions: L_s1_ = 5.5 mm; L_s2_ = 2 mm; W_s1_ = 4.5 mm; W_s2_ = 1 mm; L_sub_ = 15 mm); (**b**) simulation set up of unit cell; (**c**) simulated reflection phase of the MTS unit cell on PLA and plastic substrates for low GHz band and (**d**) for mmWave band.

The metasurface unit cell design in Figure 2a features slot-loaded Jerusalem-crossed elements with a subwavelength size, confirming its classification as a metasurface unit cell. The structure is placed on the PLA substrate with 100% infill and then on the conventional plastic substrate. To characterize the performance of the designed unit cell, simulations were conducted in Ansys HFSS. Considering Figure 2b, periodic boundary conditions were applied along the x and y axes to simulate an infinite array, while the z-direction remained open to free space. On the gray surface of the cuboid in Figure 2b, a perfect electric conductor (PEC) boundary condition is applied, ensuring zero electric field. The front and back sides represent perfect magnetic conductor (PMC) boundaries, where the magnetic field is zero. Simulating a single metasurface unit cell requires modeling it as part of an infinite array to capture its periodic nature accurately. By applying PEC and PMC boundaries, the simulation mimics an infinite array, reducing computational load while maintaining accurate performance analysis. A plane wave was incident on the unit cell from the Z_max_ port. The Floquet port mode was employed for both cases to accurately model the periodic boundary conditions and determine the unit cell’s response, as shown in Figure 2b. The comparison of the simulated phase response of the metasurface for both substrates is presented in Figure 2c,d.

Both unit cells were simulated across two frequency ranges, 2–8 GHz (low GHz band) and 22–28 GHz (mmWave band), covering key frequency bands for 5G communication. The results of the plastic cover demonstrate a wide −90° to 90° phase variation bandwidth for both frequency ranges, spanning from 2 GHz to 5.8 GHz in the low GHz band and from 22 GHz to 26 GHz in the mmWave band, whereas the PLA substrate shows a bandwidth from 2 GHz to 6.2 GHz in the low GHz band and from 22 GHz to 26.2 GHz in the mmWave band. This wide bandwidth of −90° to 90° phase shift in both frequency bands enables efficient redirection of the transmitted energy. This characteristic is particularly beneficial for wireless applications, enabling multi-band operation and improved data rates.

### 3.2. Integration of MTS with Six-Port MIMO Antenna

**Figure 3:**
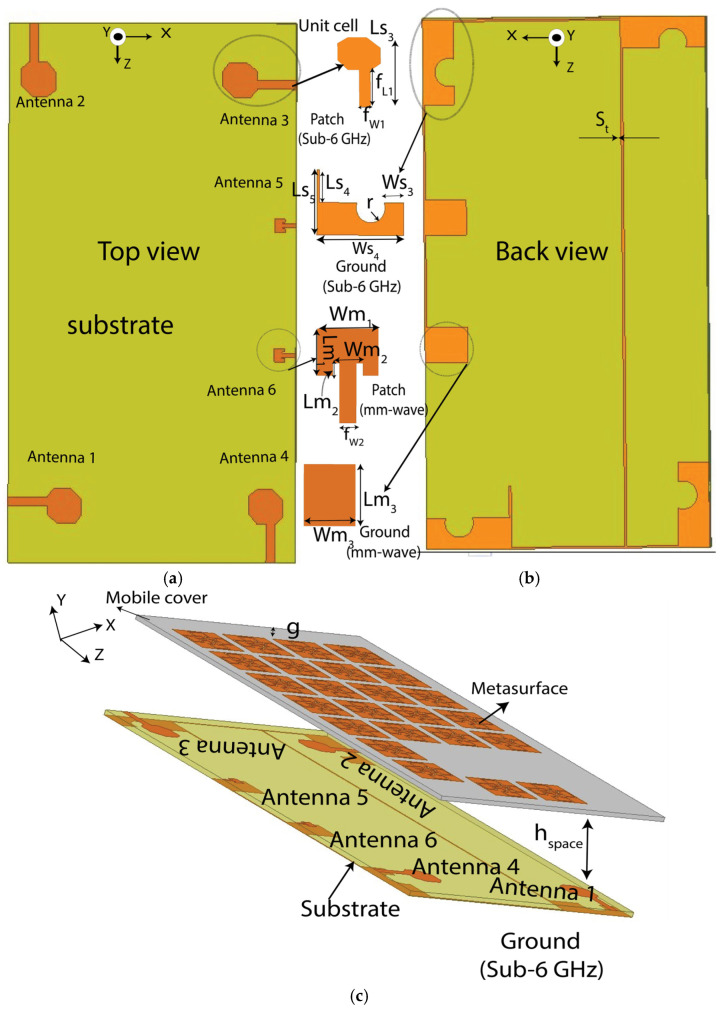
Illustration of the proposed integrated antenna: (**a**) top view, (**b**) back view, and (**c**) perspective view with metasurface on biodegradable cover. All dimensions are in millimeters: L_m1_ = 2.9; L_m2_ = 0.8; L_s3_ = 21; L_s4_ = 14.6; L_s5_ = 23.4; W_m1_ = 3.65; W_m2_ = 1.8; W_s3_ = 5.46; W_s4_ = 24; r = 3.5; f_L1_ = 10; f_L2_ = 3.8; f_w1_ = 2.9; f_w2_ = 10; g = 8 and S_t_ = 0.5; h_space_ = 5. The orange color represents the copper layer, off-white represents the PLA substrate, and yellow-green represents the FR4 substrate.

**Figure 4:**
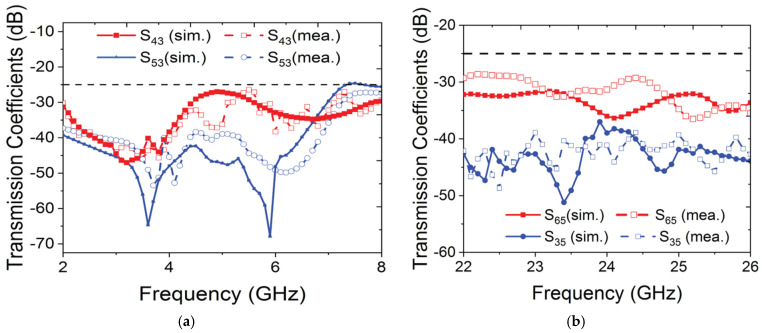
Isolation between the ports of the six-port MIMO antenna for (**a**) low GHz band and (**b**) mmWave band.

To demonstrate the efficacy of the proposed biodegradable cover for mobile applications, it is attached to the six-port MIMO antenna structure, as shown in Figure 3. The top side of the antenna consists of six radiating elements. Four of these elements (Antennas 1 to 4) are designated for low GHz frequencies, while the remaining two elements (Antennas 5 to 6) are intended for mmWave band applications. Slot loaded partial ground structures with connecting lines are utilized as a ground plane for the low GHz band, whereas full ground structures are used for the mmWave band. The entire antenna configuration is built on an FR4 substrate with a thickness of 1.6 mm, a dielectric permittivity (*ε_r_*) of 4.4, and loss tangent (*tan δ*) of 0.02. The substrate is sized at 170 mm × 80 mm to accommodate the antenna on the printed circuit board commonly used in commercial smartphones. To enhance the impedance bandwidth of the antenna, hexagonal patch, inset feed, and asymmetrical feed configurations are used, while proper impedance matching is ensured. The ground structures of all radiating elements are connected, and all dimensional parameters are optimized. The key advantage of this design is that, despite using different radiating elements for various frequency bands in a limited space, all the antennas with a common ground plane can operate simultaneously with minimal coupling between them, as shown in Figure 4. In both bands, the isolation is greater than 25 dB between the neighboring radiating elements. After optimizing all the parameters, the proposed MTS-loaded biodegradable mobile back cover was positioned at an optimized distance of *h_space_* = 5 mm to the main antenna configuration, accounting for the thickness of a typical mobile phone. This proposed mobile back cover directs most of the radiated power from the main antenna towards the backside of the mobile phone due to coupling. This results in an increase in the antenna directivity and a reduction in the back lobe.

**Figure 5:**
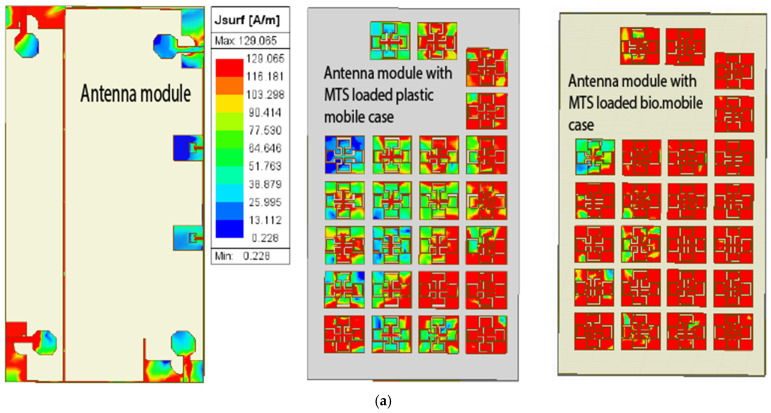

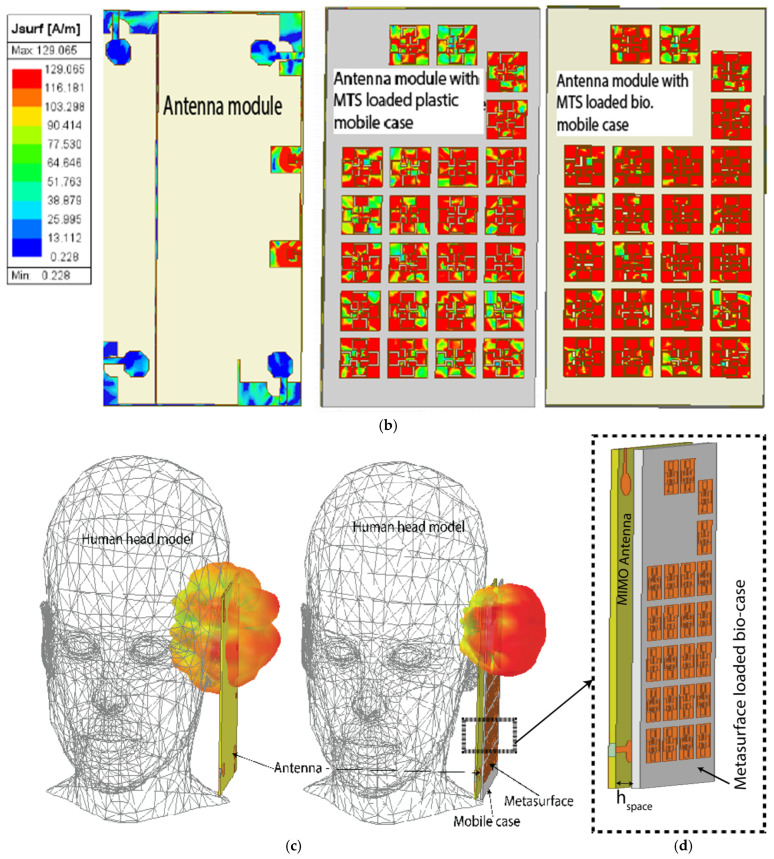
Comparison of the current distribution on different MTS-loaded covers for (**a**) low GHz and (**b**) mmWave bands and comparison of simulated 3D directivity with a dummy human head model: (**c**) without and (**d**) with the MTS-loaded biodegradable cover.

Figure 5a,b show the current distribution on the back cover for both materials across the frequency bands, while c and d illustrate the radiation patterns of the antenna with and without the proposed back cover in the vicinity of a head model. Figure 5a,b show that the surface current is well distributed over the MTS-loaded back cover, leading to an enhanced radiating area of the antenna configuration, resulting in enhanced directivity in both frequency bands.

**Figure 6:**
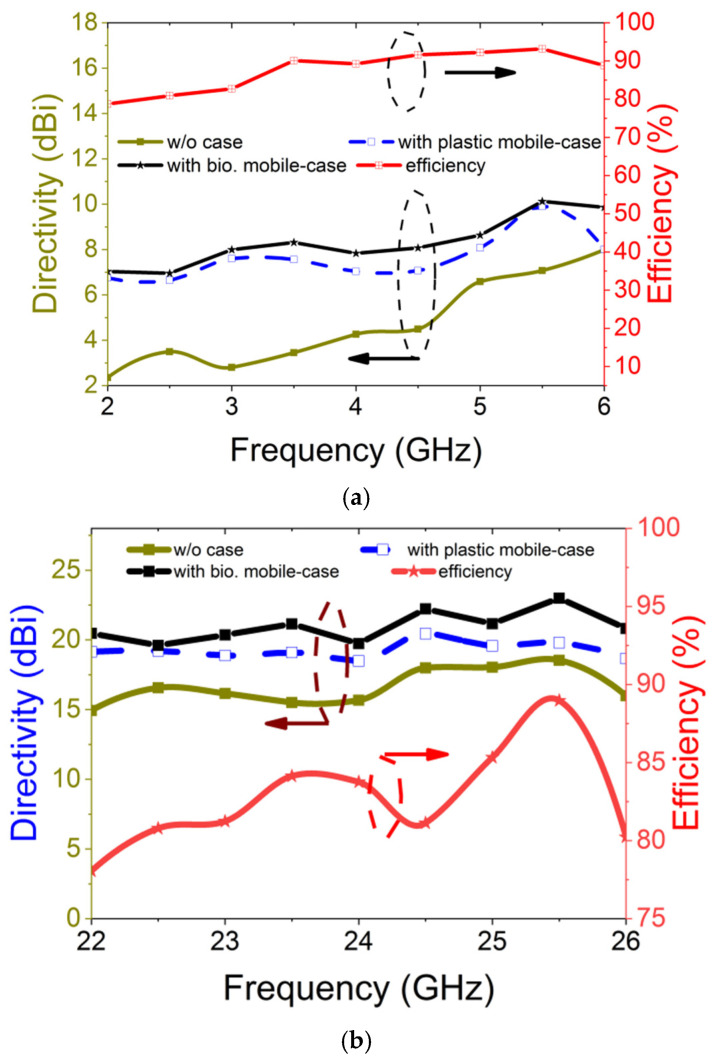
Directivity and radiation efficiency of integrated antenna with MTS-loaded biodegradable mobile cover at (**a**) low GHz band and (**b**) mmWave band.

Figure 6 illustrates the directivity of the antenna in three configurations: without a mobile back cover, with an MTS-loaded plastic back cover, and with the proposed MTS-loaded biodegradable back cover. It also presents the antenna efficiency for the proposed cover across both operating frequency bands. The antenna achieves a maximum directivity of 10.12 dBi in the low GHz band and 22.99 dBi in the mmWave band. Compared to the MTS-loaded cover on a conventional plastic substrate, the proposed design improves directivity by approximately 0.22 dBi in the low GHz band and 2.55 dBi in the mmWave band. The radiation efficiency at the lower GHz and mmWave bands is 93% and 88%, respectively, which falls within the acceptable range for practical applications.

### 3.3. Safety Study of Proposed Antenna Module for Mobile Applications

**Figure 7:**
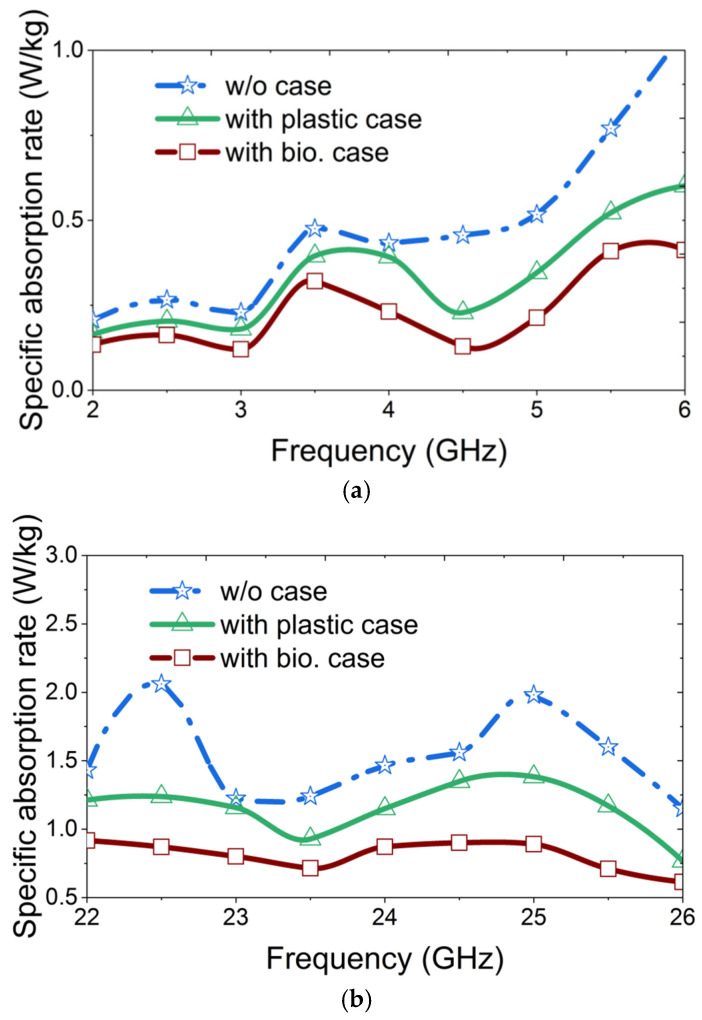
Simulated SAR of the integrated antenna module with the proposed MTS-loaded biodegradable mobile cover on the human head model at (**a**) low GHz band and (**b**) mmWave band.

**Figure 8:**
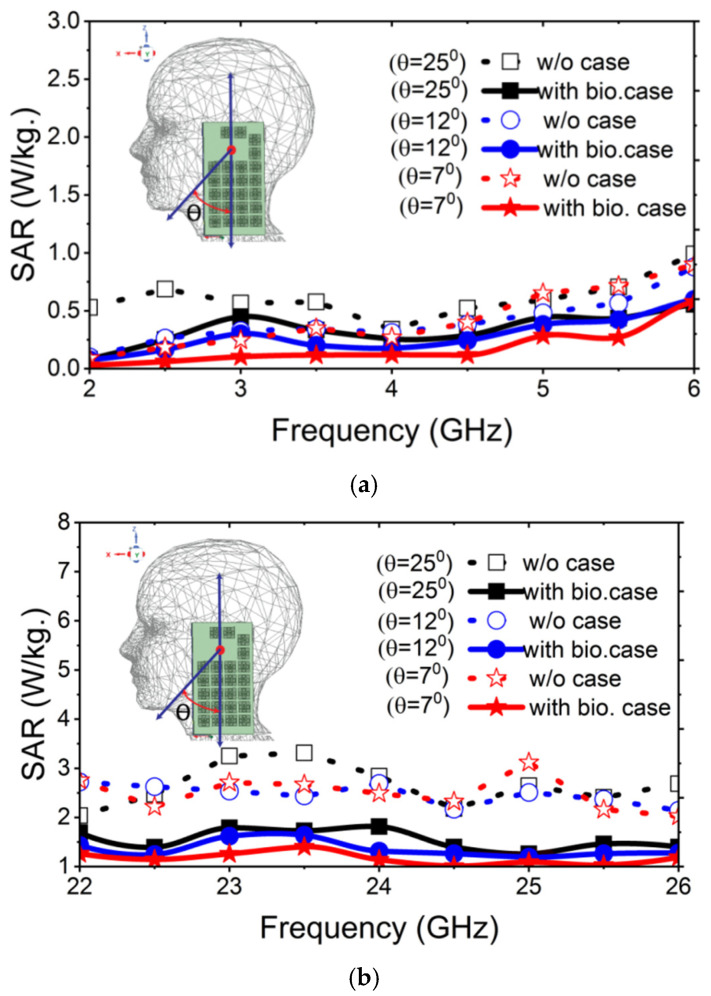
Comparison of simulated SAR for various mobile phone positions (**a**) in the low GHz band and (**b**) in the mmWave band.

Mobile phones emit electromagnetic signals during activities such as talking, texting, streaming videos or music, and even in standby mode. Given the proximity of these devices to users, it is important to evaluate the exposure of the head and body to electromagnetic radiation. In the context of 5G technology, analyzing the specific absorption rate (SAR) using a human head model is essential for ensuring compliance with safety standards. A human head model available in Ansys HFSS is utilized to evaluate the SAR performance of the antenna under three conditions: bare antenna, antenna with MTS-loaded plastic mobile cover, and antenna with MTS-loaded biodegradable cover. The IEEE and ICNIRP standards for human exposure to electromagnetic waves specify that SAR value must not exceed 1.6 W/kg for 1 g of tissue [24]. Figure 7 shows the simulated SAR values for both operating bands under three different conditions. It shows that when the antenna is excited, the maximum SAR values in the low GHz band and mmWave band are 1.06 W/kg and 2.061 W/kg, respectively. These values drop down to 0.601 W/kg and 1.238 W/kg, respectively, with the addition of the MTS-loaded plastic mobile cover. The values reduce further to 0.412 w/kg and 0.917 w/kg with the MTS-loaded biodegradable mobile cover. Therefore, with the proposed MTS-loaded cover, the maximum SAR is reduced by 61.13% in the low GHz band and 55.5% in the mmWave band. According to the standard power limit, all antennas are excited with an input power of 25 dBm in the low GHz band and 30 dBm in the mmWave band [47]. All the SAR values are assessed with zero distance from the ear position of the head model. Since the position of a mobile phone can vary under different usage conditions, the SAR value was evaluated at various tilt angles, as illustrated in Figure 7. The center of the rotational axis is indicated by the red dot in the inset of Figure 8. This variation is checked for the rotational angles of 7°, 12° and 25°. For all angle variations, higher SAR values are generally observed at larger tilt angles. This occurs because, at greater rotational positions, a larger number of antenna elements are positioned closer to the human head, leading to an increased SAR value. However, a significant reduction in SAR is achieved due to the integration of the proposed mobile back cover for both frequency bands under any angular position of the mobile phone. To the best of our knowledge, this is the first instance where biodegradable material is proposed for wireless applications, aiming not only to enhance antenna bandwidth but also to simultaneously reduce SAR across low GHz and mmWave frequency bands.

### 3.4. Measurement Results of the MIMO Antenna with the MTS-Loaded Mobile Cover

**Figure 9:**
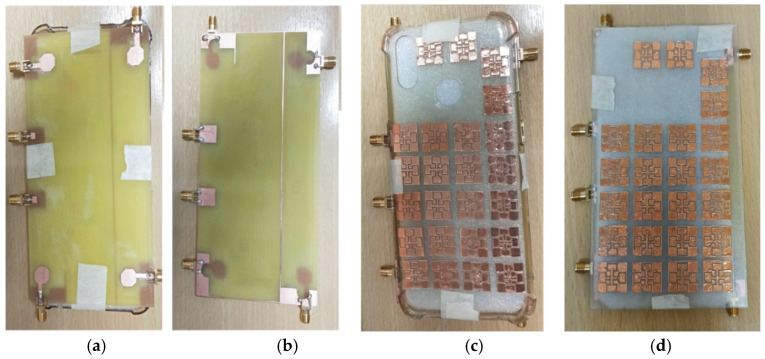
Fabricated antenna prototype: (**a**) top view, (**b**) back view, and antenna integrated with MTS fabricated on (**c**) conventional plastic cover and (**d**) proposed biodegradable cover.

**Figure 10:**
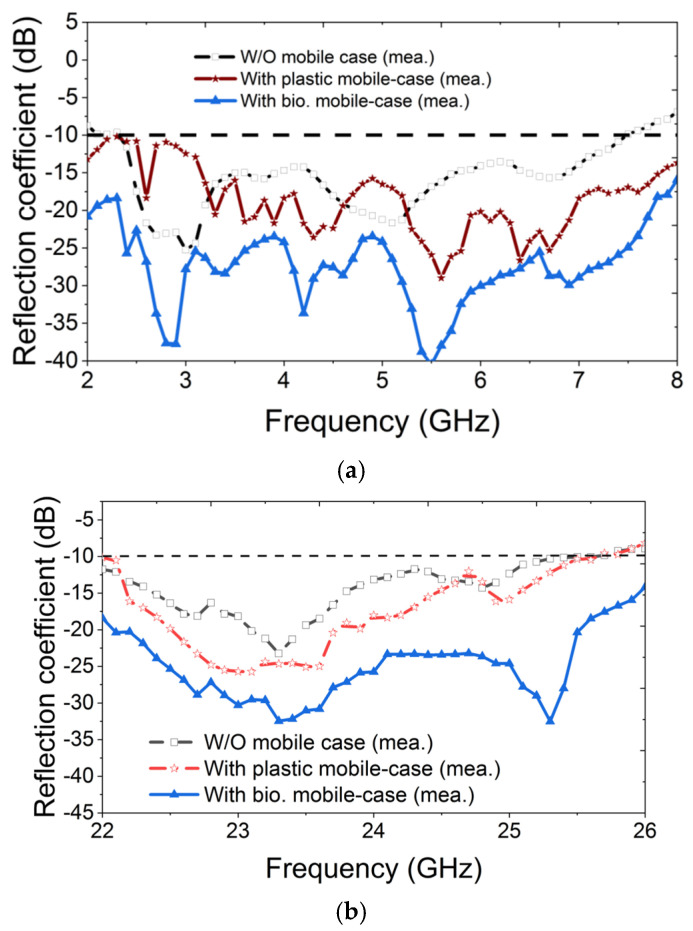
Measured S-parameters of the antenna module with and without the MTS-loaded cover in (**a**) low GHz band and (**b**) mmWave band.

Figure 9a,b show the top and back view of the fabricated antenna module on a low-cost FR4 substrate, fed by six SMA connectors. Figure 9c,d present the antenna module with an MTS-loaded plastic cover and the proposed MTS-loaded biodegradable cover. The MTS structures were fabricated using a cutting machine from Brother^TM^, Bangkok, Thailand. Figure 9a–c show the comparison of simulated and measured results for both the frequency bands. An N5242A PNA-X network analyzer was used to measure the S-parameters. Figure 10a shows that the six-port MIMO antenna operates in the frequency range of 2.4–7.4 GHz, whereas the MTS-loaded plastic cover shows a −10 dB impedance bandwidth from 2 to 8 GHz. It is evident that the use of MTS-loaded biodegradable back cover not only enhances the −10 dB bandwidth (<2 GHz to >8 GHz) but also provides better impedance matching throughout the operating bands compared to other antenna variations.

**Figure 11:**
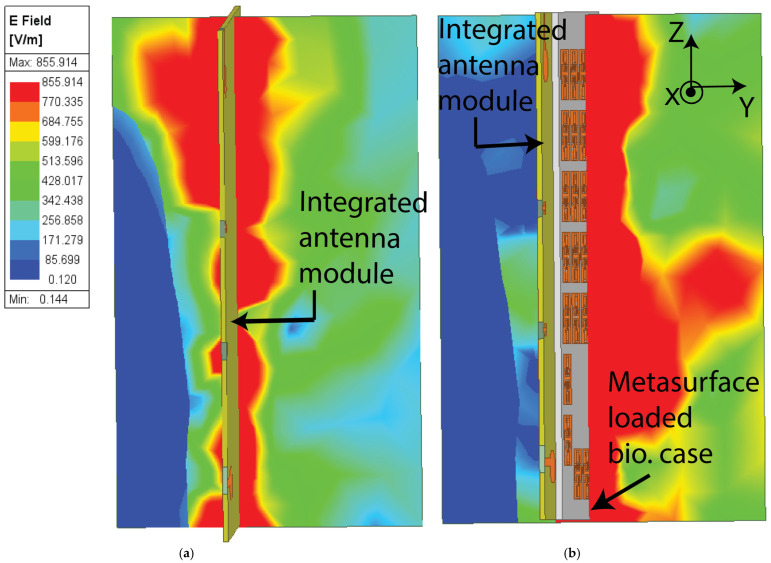
E-field distribution of the antenna (**a**) without and (**b**) with the proposed biodegradable cover.

Similarly, the antenna combined with the MTS-loaded plastic cover shows a bandwidth of 3.6 GHz from 22 GHz to 25.6 GHz in the mmWave. A 300 MHz bandwidth enhancement is obtained with the use of the MTS-loaded biodegradable cover with an improved impedance bandwidth throughout the operational bandwidth, as shown in Figure 10b. Therefore, the proposed MTS-loaded biodegradable cover increases the operating bandwidth of the antenna by 20% in the low GHz band and 8.35% in the mmWave band. Figure 11 shows the comparison of electric field distribution with and without the MTS-loaded biodegradable cover. Figure 11a demonstrates that the E-field intensity is comparable on both sides without the proposed cover. However, with the MTS-loaded biodegradable cover, the E-field intensity increases on the metasurface side, while it is significantly reduced in the opposite direction, as shown in Figure 11b. As the E-field intensity decreases in the direction opposite to the proposed cover, the SAR value in the human head model is correspondingly reduced.

**Figure 12:**
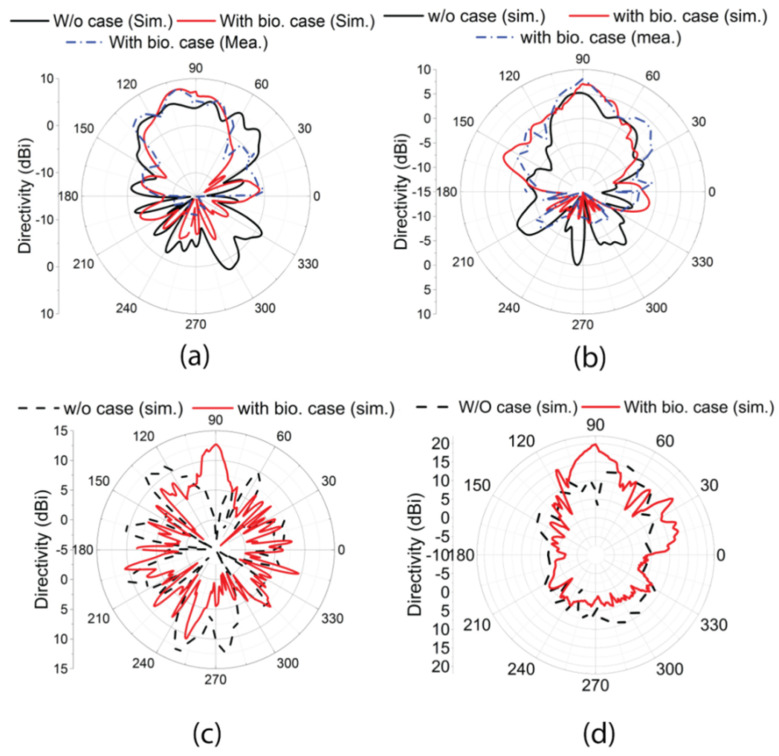
Radiation patterns of the antenna configuration with the proposed biodegradable cover at 4.5 GHz (**a**) Φ = 0° and (**b**) Φ = 90°, and at 24 GHz at (**c**) Φ = 0° and (**d**) Φ = 90°.

Figure 12 shows the radiation pattern of the antenna with and without the proposed MTS-loaded biodegradable cover in both planes and frequency bands. Due to the identical structure of the radiating elements for low GHz and mmWave bands, the radiation patterns of a single antenna element of both bands are shown. Figure 12a,b compare the radiation patterns at the azimuthal angles Φ = 0° and 90° at the frequency of 4.5 GHz. The radiation pattern of the directivity in the low GHz band was measured in the antenna test range (anechoic chamber) of the High Frequency Systems Laboratory, the Sirindhorn International Thai-German Graduate School of Engineering, King Mongkut’s University of Technology North Bangkok in Bangkok, Thailand. It shows that the proposed back cover increases the directivity by 1.45 dBi at Φ = 0° and 2.89 dBi at Φ = 90°, along with a significant reduction in the back lobe by 6.4 dB and 7.52 dB in both planes. Similarly, Figure 12c,d show the simulated radiation patterns in both planes at 24 GHz. It shows that the biodegradable cover increases the directivity by 15.55 dBi at Φ = 0° and 11.9 dBi at Φ = 90°, along with a significant reduction in the back lobe by 5.6 dB and 3.03 dB in both planes. Measurement results are not provided due to the limitation of the measurement facility. A directive radiation pattern is observed in all cases in the presence of the proposed mobile cover.

## 4. Conclusions

This work proposes a PLA-based biodegradable MTS-loaded mobile back cover to enhance the performance of an MIMO antenna system for sub-6 GHz and mm-wave bands. The integration of this biodegradable cover with the proposed antenna system effectively reduces the specific absorption rate (SAR) in the human head while enhancing the bandwidth of mobile antennas in the low GHz (2–8 GHz) and millimeter-wave (22–25.6 GHz) frequency bands. Comparative analysis shows that, while the MTS-loaded plastic cover reduces SAR from 1.06 W/kg to 0.601 W/kg in the low GHz band and from 2.061 W/kg to 1.238 W/kg in the mmWave band, the MTS-loaded biodegradable cover achieves even greater reductions, lowering SAR to 0.412 W/kg and 0.917 W/kg in the lower GHz and mmWave band, respectively. In addition to reducing SAR, the proposed cover improves antenna performance, enhancing operating bandwidth by 20% in the low GHz band and 8.35% in the mmWave band, with noticeable improvements in impedance matching. The developed metasurface-loaded cover redirects electromagnetic waves away from the human head, resulting in an increase of 5.05 dB in the lower band and 4.45 dB in the upper band. This innovative approach not only advances antenna performance but also introduces an eco-friendly alternative for mobile phone covers, providing mutual benefits for consumers and the environment.

Despite its advantages, the proposed biodegradable mobile cover has certain limitations, such as requiring specific conditions for complete degradation and reduced transparency, which may restrict its applications. Future studies could investigate various metasurface shapes on alternative biodegradable materials, such as Polybutylene Adipate Terephthalate (PBAT) blended with natural reinforcements like bamboo fibers or flax shive combined with plant-based biopolymers. These alternatives offer improved degradability and transparency compared to PLA. Additionally, the brittleness of PLA could be mitigated by developing PLA-based composites with eco-friendly additives to enhance its ductility, expanding its potential applications in antenna systems.

## Data Availability

The original contributions presented in this study are included in the article. Further inquiries can be directed to the corresponding author.

## References

[1] Cao Y., Uhrich K.E. (2019). Biodegradable and biocompatible polymers for electronic applications: A review. J. Bioact. Compat. Polym..

[2] Lawal U., Valapa R.B., Sapuan S.M., Ilyas R.A. (2021). Bioplastics: An Introduction to the Role of Eco-Friendly Alternative Plastics in Sustainable Packaging. Bio-Based Packaging: Material, Environmental and Economic Aspects.

[3] Wu Y., Gao X., Wu J., Zhou T., Nguyen T.T., Wang Y. (2023). Biodegradable Polylactic Acid and Its Composites: Characteristics, Processing, and Sustainable Applications in Sports. Polymers.

[4] Morão A., de Bie F. (2019). Life Cycle Impact Assessment of Polylactic Acid (PLA) Produced from Sugarcane in Thailand. J. Polym. Environ..

[5] Li X., Lin Y., Liu M., Meng L., Li C. (2023). A Review of Research and Application of Polylactic Acid Composites. J. Appl. Polym. Sci..

[6] Khouri N.G., Bahú J.O., Blanco-Llamero C., Severino P., Concha V.O.C., Souto E.B. (2024). Polylactic Acid (PLA): Properties, Synthesis, and Biomedical Applications–A Review of the Literature. J. Mol. Struct..

[7] Freeland B., McCarthy E., Balakrishnan R., Fahy S., Boland A., Rochfort K.D., Dabros M., Marti R., Kelleher S.M., Gaughran J. (2022). A Review of Polylactic Acid as a Replacement Material for Single-Use Laboratory Components. Materials.

[8] Raquez J.-M., Habibi Y., Murariu M., Dubois P. (2013). Polylactide (PLA)-based nanocomposites. Prog. Polym. Sci..

[9] Rangan S., Rappaport T.S., Erkip E. (2014). Millimeter-Wave Cellular Wireless Networks: Potentials and Challenges. Proc. IEEE.

[10] Zhang X., Tan T.-Y., Wu Q.-S., Zhu L., Zhong S., Yuan T. (2021). Pin-Loaded Patch Antenna Fed With a Dual-Mode SIW Resonator for Bandwidth Enhancement and Stable High Gain. IEEE Antennas Wirel. Propag. Lett..

[11] Jeong G.-T., Kim W.-S., Kwak K. (2012). Dual-Band Wi-Fi Antenna with a Ground Stub for Bandwidth Enhancement. IEEE Antennas Wirel. Propag. Lett..

[12] Liu G., Hu P., Su G., Pan Y. (2023). Bandwidth and Gain Enhancement of a Single-Layer Filtering Patch Antenna Using Reshaped TM12 Mode. IEEE Antennas Wirel. Propag. Lett..

[13] Xu K.-D., Xu H., Liu Y., Li J., Liu Q. (2018). Microstrip Patch Antennas with Multiple Parasitic Patches and Shorting Vias For Bandwidth Enhancement. IEEE Access.

[14] Zhou J., Cai J., Chen J.-X. (2024). Contactless Varactor-Loaded Bandwidth-Enhanced Frequency-Reconfigurable Patch Antenna. IEEE AWPL.

[15] Arif A., Zubair M., Ali M., Khan M.U., Mehmood M.Q. (2019). A Compact, Low-Profile Fractal Antenna for Wearable On-Body WBAN Applications. IEEE Antennas Wirel. Propag. Lett..

[16] Zada M., Shah I.A., Yoo H. (2020). Metamaterial-Loaded Compact High-Gain Dual-Band Circularly Polarized Implantable Antenna System for Multiple Biomedical Applications. IEEE Trans. Antennas Propag..

[17] Fang Y., Jia Y., Zhu J.-Q., Liu Y., An J. (2024). Self-Decoupling, Shared-Aperture, Eight-Antenna MIMO Array With MIMO-SAR Reduction. IEEE Trans. Antennas Propag..

[18] Lu B., Pang B., Hu W., Jiang W. (2021). Low-SAR Antenna Design and Implementation for Mobile Phone Applications. IEEE Access.

[19] Kwak S.-I., Sim D.-U., Kwon J.H. (2011). Design of Optimized Multilayer PIFA With the EBG Structure for SAR Reduction in Mobile Applications. IEEE Trans. Electromagn. Compat..

[20] Varheenmaa H., Ylä-Oijala P., Lehtovuori A., Viikari V. (2022). SAR Reduction With Antenna Cluster Technique. IEEE Trans. Antennas Propag..

[21] Yang C., Yao Y., Yu J., Chen X. (2012). Novel Compact Multiband MIMO Antenna for Mobile Terminal. Int. J. Antennas Propag..

[22] Wong K.-L., Hsu Y.-H., Lee C.-Y., Li W.-Y. (2024). Wideband 4-Port Patch Antenna Module Based Compact 8-Port Two-Module Antenna for 6G Upper Mid-Band 8 × 4 Device MIMO With Enhanced Spectral Efficiency. IEEE Access.

[23] Liu D.Q., Zhang M., Luo H.J., Wen H.L., Wang J. (2018). Dual-Band Platform-Free PIFA for 5G MIMO Application of Mobile Devices. IEEE Trans. Antennas Propag..

[24] (2006). IEEE Standard for Safety Levels with Respect to Human Exposure to Radio Frequency Electromagnetic Fields, 3 kHz to 300 GHz.

[25] ICNIRP (1998). Guidelines for limiting exposure to time-varying electric, magnetic and electromagnetic fields (up to 300 GHz). Health Phys..

[26] Yadav H., Rai U., Singh R. (2021). Radiofrequency radiation: A possible threat to male fertility. Reprod. Toxicol..

[27] Durusoy R., Hassoy H., Özkurt A., Karababa A.O. (2017). Hassoy Mobile phone use, school electromagnetic field levels and related symptoms: A cross-sectional survey among 2150 high school students in Izmir. Environ. Health.

[28] Shin J.C., Kim J., Grigsby-Toussaint D. (2017). Mobile phone interventions for sleep disorders and sleep quality: Systematic review. JMIR Mhealth Uhealth.

[29] Zhang T., Pang X., Zhang H., Zheng Q. (2023). Ultrabroadband RCS Reduction and Gain Enhancement of Patch Antennas by Phase Gradient Metasurfaces. IEEE Antennas Wirel. Propag. Lett..

[30] Zheng Q., Liu W., Zhao Q., Kong L., Ren Y.-H., Yang X.-X. (2024). Broadband RCS Reduction, Antenna Miniaturization, and Bandwidth Enhancement by Combining Reactive Impedance Surface and Polarization Conversion Metasurface. IEEE Trans. Antennas Propag..

[31] Sufian M.A., Hussain N. (2024). Metasurface-Based Phone Case for the SAR Reduction of the 5G Mobile Phones. IEEE Trans. Electromagn. Compat..

[32] Sufian M.A., Hussain N., Askari H., Park S.G., Shin K.S., Kim N. (2021). Isolation Enhancement of a Metasurface-Based MIMO Antenna Using Slots and Shorting Pins. IEEE Access.

[33] Signal Boosters (2024). Cellular frequency bands: A simple breakdown. https://www.signalboosters.com/blog/cellular-frequency-bands-a-simple-breakdown/.

[34] Dahman Y. (2012). Poly (Lactic Acid): Green and Sustainable Plastics. Ferment. Technol..

[35] Al-Tayyar N.A., Youssef A.M., Al-Hindi R. (2020). Antimicrobial food packaging based on sustainable bio-based materials for reducing foodborne pathogens: A review. Food Chem..

[36] Resende T.M., Costa M.M. (2020). Biopolymers of sugarcane. Sugarcane Biorefinery, Technology and Perspectives.

[37] Avinc O., Khoddami A. (2009). Overview of poly (lactic acid) (PLA) fibre: Part I: Production, properties, performance, environmental impact, and end-use applications of poly (lactic acid) fibres. Fibre Chem..

[38] Pirsa S., Sani I.K., Mirtalebi S.S. (2022). Nano-biocomposite based color sensors: Investigation of structure, function, and applications in intelligent food packaging. Food Packag. Shelf Life.

[39] Raja M.M., Lim P.Q., Wong Y.S., Xiong G.M., Zhang Y., Venkatraman S., Huang Y. (2019). Polymeric Nanomaterials: Methods of Preparation and Characterization. Nanocarriers for Drug Delivery.

[40] Shaikh S., Yaqoob M., Aggarwal P. (2021). An overview of biodegradable packaging in food industry. Curr. Res. Food Sci..

[41] Ashby M.F. (2012). Materials and the Environment: Eco-informed Material Choice.

[42] Handa M., Soni M., Beg S., Shukla R. (2023). Nanoscaffolds and role of 3D-printed surgical dressings for wound healing application. Nanotechnology and Regenerative Medicine.

[43] Castro-Aguirre E., Iniguez-Franco F., Samsudin H., Fang X., Auras R. (2016). Poly (lactic acid)—Mass production, processing, industrial applications, and end of life. Adv. Drug Deliv. Rev..

[44] Ahmad A., Banat F., Alsafar H., Hasan S.W. (2023). An overview of biodegradable poly (lactic acid) production from fermentative lactic acid for biomedical and bioplastic applications. Biomass Convers. Biorefinery.

[45] Latti K.-P., Kettunen M., Strom J.-P., Silventoinen P. (2007). A review of microstrip T-resonator method in determining the dielectric properties of printed circuit board materials. IEEE Trans. Instrum. Meas..

[46] Balanis C.A. (2005). Antenna Theory: Analysis and Design.

[47] Islam S., Zada M., Yoo H. (2022). Highly Compact Integrated Sub-6 GHz and Millimeter-Wave Band Antenna Array for 5G Smartphone Communications. IEEE Trans. Antennas Propag..

